# hCG Triggering in ART: An Evolutionary Concept

**DOI:** 10.3390/ijms18051075

**Published:** 2017-05-17

**Authors:** Anat Hershko Klement, Adrian Shulman

**Affiliations:** 1 Department of Obstetrics and Gynecology, Meir Medical Center, Kfar Saba 4428164, Israel; Adrian.Shulman@clalit.org.il; 2 The Sackler School of Medicine, Tel Aviv University, Tel Aviv-Yafo 6997801, Israel

**Keywords:** chorionic gonadotropin, LH receptor, ovulation induction, ovarian stimulation

## Abstract

Human chorionic gonadotropin (hCG) is no longer a single, omnipotent ovulation triggering option. Gonadotropin releasing hormone (GnRH) agonist, initially presented as a substitute for hCG, has led to a new era of administering GnRH agonist followed by hCG triggering. According to this new concept, GnRH agonist enables successful ovum maturation, while hCG supports the luteal phase and pregnancy until placental shift.

## 1. Introduction

The physiological luteinizing hormone (LH) surge, well-known as the crucial step in ovum meiosis and maturation, as well as in maturation of its supporting cells, has been traditionally replaced by human chorionic gonadotropin (hCG) in artificial reproductive technologies (ART) cycles. The actual event of ovulation is far from simple and involves multiple cascades and processes [1,2,3,4], most of which are still poorly understood [5]. We should also consider that this LH surge is not the sole surge in normal physiological ovulatory cycles. The follicle stimulating hormone (FSH) surge, which follows the LH-induced progesterone rise, is a surrogate surge involving plasminogen activators. It assures adequate LH receptoral response in the granulosa cells [2]. After hCG became the substitute in this phase, its role was extended to the follicular phase [6], where the long-standing debate on the role of LH/hCG has not yet been resolved [6,7,8].

We thoroughly understand that both LH and hCG bind to the same LH/hCG receptor (LHCGR), but this does not imply an identical response. The major structural difference between the two hormones is the sequence of the b-subunit and the critical difference is their pharmacokinetics; hence, clearance [9]. hCG has a slower plasma metabolic clearance, which consists of a rapid phase in the first 5 to 9 h following its administration and a slower phase in the 1 to 1.3 days after administration. After 36 h, the calculated half-life of hCG is 2.32 days, as compared with LH, for which estimates have ranged from 1 h [3] to 3 to 5 h [9]. Even in terms of receptoral exposure, one cannot infer the same behavior, since the natural mid-cycle surge is characterized by three phases lasting a total of 48 h [10], while the induced surge follows a different pattern.

The equivalence of human recombinant LH and human recombinant hCG has been studied thoroughly in terms of potency, kinetics and response [11]. In vitro models found significant differences in the dominance of the intracellular cascade pathways (such as AKT, ERK 1/2 and PKA), and a clear five-fold potency for hCG and a significantly different time-response. As supported by other groups [11,12,13], the downstream effects of hLH and hCG differ. Therefore, their equivalence is challenged. Reflecting their physiological roles, current knowledge generally depicts a high steroidogenic potential for hCG, whereas LH is a more proliferative, anti-apoptotic agent [14]. Simoni’s group has also questioned the biological differences between natural hCG/LH and the artificial components, which demonstrate comparable intracellular responses when gonadotropin extracts versus recombinants are used [11,14]. This is an important notion, because natural gonadotropins are a heterogeneous group, with different components (i.e., sialic acid content and glycosylation weight). Although injectable gonadotropins could contain a mixture of molecules, they are not necessarily identical to the natural forms. A 2016 Cochrane review [15] also discussed the same question by testing the clinical aspects. When urinary hCG was compared to the recombinant form, no statistically significant differences were detected in terms of pregnancy rate, live birth rate, ovarian hyper-stimulation syndrome (OHSS) and miscarriage rate.

We should also keep in mind that medicated cycles change the biological pattern, not only in terms of gonadotropins, but also in terms of the LHCGR. This receptor has a dynamic expression pattern throughout the cycle. It reaches maximum expression and effect in the mid-luteal phase and decreases with corpus luteum regression [16]. In contrast, medicated cycles are characterized by peak biological activity at mid-cycle and lowest activity during the luteal phase. This is another potential explanation for the differences in LH/hCG mediated cascades.

From a clinical perspective, the most troubling issue arising from the use of hCG for final follicular maturation is ovarian hyperstimulation syndrome (OHSS), because of its prolonged clearance. hCG is considered fundamental in triggering OHSS due to its ability to up-regulate VEGF expression in luteinized granulosa cells [17]. VEGF is already elevated during the gonadotropin stimulation phase, preceding hCG injection. However, it is further stimulated by hCG administration and can be found in the corpus luteum vessels and throughout the corpus luteum [18]. VEGF is considered the major driving force in the hyper-permeability characterizing OHSS and is therefore a key player in its pathophysiology.

Is hCG alone, without previous ovarian stimulation, sufficient to explain the pathophysiology of OHSS? This interesting concept was tested in unstimulated pregnancies with hCG concentrations above 150,000 IU/L. None of the patients developed spontaneous OHSS [19]. The literature provides a few anecdotal cases of spontaneous OHSS, which are characterized by familial background, and recur in subsequent pregnancies [20,21]. Others have described FSH receptor mutations as a mechanism that explains some of the cases presenting with OHSS [22,23]. Therefore, it seems that the general background conditions for OHSS should include multiple corpora lutea, which respond to hCG and lead to very large increases in VEGF production and VEGF receptivity.

If indeed such distinctive intracellular events follow LHCGR activation by hCG compared to LH, and if hCG is closely related to the occurrence of OHSS, why is recombinant LH (rLH) not commonly used for final ovarian maturation? In a multicenter study, patients received either rhLH or u-hCG to achieve final follicular maturation [9]. The rhLH doses were 5000, 15,000, 30,000, or 15,000 + 10,000 IU (second injection administered 3 days after the first, and u-hCG was consistently 5000 IU). Although not statistically significant, the hCG group seemed to perform better and the lowest rhLH dose was suboptimal when compared with the higher dose. The 15,000 IU to 30,000 IU dose of rhLH provided the highest efficacy-to-safety ratio. These very high doses of injectable rLH required to achieve sufficient biological effect raise serious cost vs. efficacy concerns and explain why recombinant LH has not become the default ovulation triggering option. A 2016 Cochrane analysis found that the quality of evidence regarding the rLH performance was very low, which strongly limits the ability to draw any conclusions regarding its use for triggering, even when ignoring cost issues [15]. Troubling information regarding reduced clinical pregnancy rates in the rLH group further restricts the use of this medication for ovulation triggering [9]. These reasons explain why GnRH agonist triggering in a GnRH antagonist cycle has been suggested and used as an option for triggering ovulation. It can be a preventive measure against OHSS, especially when combined with an efficient freeze/thaw system.

ART medicine has entered a new era of zero tolerance to OHSS, otherwise termed an “OHSS free clinic” [24,25]. In the current ART era, the use of hCG triggering is challenged as a fundamental element in medical therapy. Multiple international clinics have reported routine, successful use of GnRH agonist triggering, to the point where it seems that hCG will be reserved for special circumstances or for in vivo fertility cycles. GnRH agonist triggering has been proven, to the highest level of evidence, as protective from OHSS [26], although it is still inferior in terms of pregnancy rate and live births [26]. The superiority of GnRH agonist triggering is clear in terms of oocyte donors, women or units avoiding fresh transfers (for whatever reasons) and in the context of fertility preservation [26].

Can we explain why pregnancy rates are compromised after the use of GnRH agonist triggering? This is a surprising observation, since one would not expect endogenous LH and FSH surges to be so inferior to hCG triggering. It is even more puzzling when we consider that in non-suppressed in vivo cycles, circulating estrogen and progesterone levels were found sufficient [27] and almost identical when these triggering options were compared. Moreover, pregnancy rates were similar [27,28]. A possible explanation can arise from the LH/hCG receptor in the reproductive tract along with the prolonged half-life of hCG as compared to LH. Possible effects of hCG on non-gonadal tissues and especially on endometrial cells could explain the role of hCG in promoting pregnancy [29]. An example of the supportive effects of hCG was published in a randomized clinical trial, where intrauterine infusion of HCG prior to embryo transfer was shown to increase implantation and pregnancy rates [30].

The use of GnRH agonist triggering has raised interest in a new concept: dual triggering, which combines both hCG and endogenous LH/FSH surges. The idea of combining hCG with GnRH agonist triggering was introduced in 2008 in an effort to reduce the increased pregnancy loss rate associated with GnRH agonist triggering [31]. It was suggested that its use, even as a “substituting dose”, can aid in ovum maturation, provide sustained support and supplement the surge with endogenous LH [31]. Following introduction of the “dual triggering concept”, other indications were published, such as successful dual triggering for empty follicle syndrome [32] and for improving the yield of oocytes in patients with a low oocyte/follicle ratio [33]. It seems that GnRH agonist triggering has become established as an efficient alternative for egg maturation, while hCG provides sustained support for the luteal phase. In other words, the exact formulation of luteal phase support can match the relative advantage of hCG, while still offering the benefits of GnRH agonist triggering. From this perspective, we can anticipate new studies testing different dosage combinations of GnRH agonist and hCG, enabling us to tailor the correct ovulation trigger for a specific patient [34].

Does hCG have a role in triggering ovulation of small follicles that were traditionally considered “immature”? The concept of in vitro maturation (IVM) has provided surprisingly interesting and challenging information: hCG priming of follicles smaller than 10 mm in diameter resulted in similar MII oocyte yields compared to follicles larger than 10 mm [35]. Some argue that the term IVM should be reserved for cycles completely without gonadotropic exposure and where the ovum matures completely in vitro [36]. This contradiction arises from the fact that opposed to the in vitro notion, some 8–12 mm follicles can respond to hCG, and reach the metaphase II (MII) stage at recovery with no need for in vitro maturation [36]. These in vivo-matured eggs retrieved in an “IVM cycle” are developmentally superior compared with those that are matured in vitro [37]. Some groups support the use of hCG in IVM cycles only when FSH priming was used; demonstrating the highest yield of MII stage oocytes for this specific combination [38]. Unsurprisingly, the use of GnRH agonist triggering has increased across ART practices, leading to a promising case report describing the successful use of GnRH agonist triggering in an IVM cycle [39]. This preliminary report reflects a possible advantage of GnRH-ag in IVM cycles, especially those performed for fertility preservation.

## 2. Summary

Until the last decade, hCG was an essential component of ART cycles. In the last few years, “the post-long protocol dominance era” has yielded a fascinating evolution of ovulation triggering modes. GnRH-ag triggering replaced hCG as a means of avoiding OHSS, and has further led the ART community into new horizons of egg maturation. In the current era, GnRH-ag triggering is being tested in combination with hCG as an option to achieve better results in special circumstances and/or populations. Combining hCG and GnRH-ag increases their advantages and is a promising area for future research. The new concept is dichotomous: GnRH-ag is the better option for egg maturation and hCG is superior for luteal phase support. Hopefully, future research will teach us the best combination and the ideal timing for this treatment method.
